# A significant gap between inadequate pharmacotherapy and substantial unmet needs in palmar hyperhidrosis management in China: insights from a questionnaire-based survey among outpatients

**DOI:** 10.3389/fphar.2025.1715189

**Published:** 2026-01-08

**Authors:** Ning Pang, Yi Liu, Chaoqun Ma, Yingkun Liu, Lin Huang, Xiaohong Zhang, Yanguo Liu

**Affiliations:** 1 Department of Pharmacy, Peking University People’s Hospital, Beijing, China; 2 Department of Pharmacy, Peking University Third Hospital, Beijing, China; 3 Department of Pharmacy, Beijing Miyun District Traditional Chinese Medicine Hospital, Beijing, China; 4 Department of Pharmacy, Affiliated Hospital of Chifeng College, Nei Mongol, China; 5 Department of Thoracic surgery, Peking University People’s Hospital, Beijing, China

**Keywords:** primary palmar hyperhidrosis, prior concerns of pharmacotherapy, questionnaire survey, treatment status, willingness of future pharmacotherapy

## Abstract

**Background:**

Palmar hyperhidrosis, characterized by excessive sweating primarily affecting the hands, significantly impairs quality of life and psychological well-being. Although topical agents, iontophoresis, microwave therapy, and sympathectomy are established interventions, real-world treatment patterns and pharmacotherapeutic adequacy among Chinese patients remain poorly delineated.

**Methods:**

An anonymous, questionnaire-based survey was conducted among consecutive patients at a tertiary thoracic surgery clinic in China between March 2023 and October 2024. A total of 363 valid responses were collected and analyzed using SPSS 27.0. Descriptive statistics and multivariable logistic regression were used to evaluate treatment patterns, patient preference, and predictors of treatment selection.

**Results:**

The study cohort was composed predominantly of individuals with severe disease (87.3% HDSS grade 3–4), and nearly all participants (97.8%) presented with palmar hyperhidrosis. Regarding treatment history, only 38.1% had previously received pharmacotherapy, while 32.6% had utilized Traditional Chinese Medicine (TCM) therapeutic approaches, including TCM, acupuncture, massage and manipulation therapies. Antiperspirants (28.6%) and TCM (25.0%) were the most frequently used prior pharmacotherapies, yet both were associated with limited therapeutic efficacy. According to our survey, owing to high symptom severity, 52.9% had a general knowledge of surgical intervention before the outpatient visit. Primary concerns regarding previous pharmacotherapy included insufficient efficacy and localized adverse effects, particularly skin irritation. Notably, 50.0% of participants remained receptive to future pharmacotherapy, showing a preference for topical formulations (29.3%) and reduced dosing frequency (31.6%). Gender and disease severity were identified as significant determinants of treatment choice. Importantly, prior negative experiences - such as inefficacy and side effects - did not significantly diminish willingness to consider future pharmacologic treatment.

**Conclusion:**

Inadequate management and suboptimal treatment outcomes represent considerable challenges in the care of palmar hyperhidrosis in China. Existing pharmacotherapeutic options are constrained by limited availability, inadequate efficacy, and a narrow range of approved agents - a reality that highlights a critical disconnect with substantial patient demand. These findings underscore an urgent need to accelerate drug development and clinical translation in this field.

## Introduction

Palmar hyperhidrosis (PH) is a focal sweating disorder triggered by abnormal sympathetic excitation that leads to sweating beyond thermoregulatory needs (Fujimoto et al., 2023). Although reported prevalence rates vary geographically, primary PH is estimated to affect approximately 5%–20% of the global population (Liu et al., 2016; Ribeiro Santos Morard et al., 2019; Wadhawa et al., 2019). An epidemiological survey in China reported a prevalence of 2.08% for PH (Lai et al., 2015). Irrespective of geographic origin, PH significantly impairs health-related quality of life and is associated with substantial psychological distress (Woo et al., 2022). Nevertheless, PH remains underdiagnosed and undertreated, with only approximately half of affected individuals seeking medical attention (Wadhawa et al., 2019).

Current therapeutic strategies follow a stepwise approach based on symptom severity and prior treatment response (McConaghy and Fosselman, 2018). First-line therapy typically involves topical antiperspirants, mainly aluminum chloride, which act by mechanically obstructing eccrine ducts. However, skin irritation frequently limits long-term adherence. Iontophoresis, which delivers electrical current through water or supplemented media, provides a non-invasive alternative but demands frequent and time-consuming sessions. Botulinum toxin A (BTX-A) injections serve as a highly effective second-line treatment through inhibition of acetylcholine release at neuromuscular junctions of sweat glands. Although BTX-A demonstrates significant reductions in sweating severity, its utility is constrained by injection-related pain, transient efficacy (generally 6–8 months), and high cost. Oral anticholinergics (e.g., glycopyrrolate, oxybutynin) are generally reserved for refractory cases but are often discontinued due to systemic adverse effects such as dry mouth, blurred vision, urinary retention, and constipation. Although some topical anticholinergic agents have been approved by the FDA, they are not accessible in China (Wong et al., 2022). For severe and treatment-resistant PH, endoscopic thoracic sympathectomy may be considered; however, this procedure carries substantial risks, including compensatory hyperhidrosis with prevalence of more than 50% in most studies, making it a measure of last resort (Du et al., 2018).

Despite the availability of clinical guidelines, significant gaps persist in characterizing real-world treatment practices and patient experiences, particularly within the Chinese PH population with severe symptom. It is anticipated that the current management is influenced by multiple factors, including disease awareness, treatment adherence, medication accessibility, and the alignment of therapies with patient needs. Nevertheless, robust real-world data on treatment patterns, satisfaction, and unmet needs remain scarce. Furthermore, the limitations of existing therapeutic options highlight a compelling need for more effective, durable, and accessible pharmacological treatments. This study employs questionnaire-based survey to elucidate the current treatment landscape among PH patients in China and to assess their actual willingness toward future pharmacotherapy.

## Materials and methods

### Questionnaire development and validation

This study is a descriptive, cross-sectional survey based on an electronic questionnaire platform (SoJump), developed following a comprehensive review of the literature on the current treatment status of PH and patient-reported outcomes. The initial draft of the questionnaire covered multiple dimensions, including demographic characteristics, disease history and severity, prior treatment experiences and their effectiveness, as well as treatment needs and concerns. To ensure the content validity of the questionnaire, we invited one clinical expert from thoracic surgery, one senior clinical pharmacist and one statistical expert to evaluate the appropriateness, relevance and completeness of the items. Based on their feedback, we revised the wording and structure of the questionnaire. Before the formal investigation, we conducted a pilot survey on 6 patients with PH who met the inclusion criteria to evaluate the surface validity, understandability and completion time required of the questionnaire. Based on the results of the pilot survey, we made final revisions to some potentially ambiguous items. We evaluated the internal consistency reliability of the questionnaire, and the Cronbach’s α coefficients for the patient-reported efficacy dimension and the adverse reaction dimension were 0.855 and 0.774 respectively, indicating that the questionnaire had qualified reliability.

### Data collection process and quality control

The survey was conducted between March 2023 and October 2024 in the thoracic surgery clinic of Peking University People’s Hospital, which is a dedicated Hyperhidrosis Clinic. The study protocol was approved by the Institutional Review Board (Approval NO.: 2023PHB116-001). Participants were consecutively recruited from outpatients with a confirmed diagnosis of primary PH by a specialist surgeon. Cases of secondary PH, whose treatment centers on addressing the underlying etiology, were excluded from the present study. This consecutive enrollment approach was employed to minimize selection bias. We obtained full informed consent from all the enrolled outpatients and had them complete the forms based on the principle of voluntary participation. We assured that the questionnaire would be filled out anonymously and the data collected would be kept confidential, strictly for this survey only.

### Definitions of key variables

Questionnaire survey required patients to assess their own condition according to the Hyperhidrosis Disease Severity Scale (HDSS). Specifically speaking, HDSS score criteria divide PH into four categories: grade 1-sweating is never noticeable and never interferes with daily activities; grade 2-sweating is tolerable but sometimes interferes with daily activities; grade 3-sweating is barely tolerable and frequently interferes with daily activities; grade 4-sweating is intolerable and always interferes with daily activities.

In the part of evaluation of willingness for future pharmacotherapy. The ‘willingness’ referred refers to the patient’s willingness to undergo pharmacotherapy. The ‘reluctance’ referred to the situation where the patients were unwilling to undergo pharmacotherapy for PH. The ‘neutral preference’ referred to the situation where patients only have a 50% likelihood of wanting to undergo drug pharmacotherapy.

### Sample size calculation and statistical analysis

Given that we will subsequently use the chi-square test to analyze the influencing factors of patients’ treatment choices, we employed the G*Power software to calculate the minimum sample size required for this research. With a medium effect size (Cohen’s d = 0.3), α = 0.05, and a power of 0.95, the calculated result indicates that the minimum sample size is 220. Finally, a total of 363 outpatients provided informed consent and were enrolled. We conducted Shapiro–Wilk tests on all continuous variables (age, weight, height and Duration of PH) and found that none followed a normal distribution (*P* = 0.000); consequently, non-parametric tests were used for all subsequent statistical analyses.

Data cleaning and processing were performed independently by two researchers. Inconsistent or contradictory responses were excluded for specific analyses, resulting in 304 retained datasets. To be specific, questions 14 (patient-reported efficacy) and 15 (adverse reaction) in Part 3 of the questionnaire collected patient-reported efficacy and adverse reactions after previous drug treatments, respectively. However, inconsistencies were found in the collected data. For example, some patients selected “not used” for a particular treatment regimen in question 14 but reported adverse reactions for the same treatment regimen in question 15 without selecting “not used”, resulting in data inconsistencies. For such ambiguous data that was difficult to interpret, we discarded it and ultimately retained 304 datasets. For missing data, such as the duration of palmar hyperhidrosis, we replaced the missing values with the mean value. In the multinomial logistic regression, 14 values (4.60%) were missing for the prior pharmacotherapy experience. Listwise deletion was applied to handle these missing values. We conducted an internal consistency reliability test on the collated questionnaire data. Statistical analyses included Chi-Square Test, Spearman’s correlation coefficient, Spearman’s rank correlation analysis, univariate analysis, and multinomial logistic regression, performed using SPSS 27.0. The complete questionnaire and participant flowchart of our study are available in the Supplementary Material.

## Results

### Baseline characteristics of the surveyed outpatients

As summarized in Table 1, a total of 363 outpatients with PH were enrolled in the study, comprising 49.9% males and 50.1% females. The median age was 27 ± 8 years, with median height and weight of 169 ± 8 cm and 63.3 ± 12.8 kg, respectively. Education background varied among participants: the majority (57.8%) held undergraduate degrees, 29.2% had junior and senior high school, 12.4% had graduate-level education, and 0.55% had primary school education or below. Occupational distribution was diverse, with students representing the largest subgroup (35.3%), followed by individuals employed in miscellaneous professions (32.8%). All other occupational categories each accounted for less than 5% of the cohort. Comorbidities were reported by 24.2% of patients, with the most prevalent being anxiety (6.06%), insomnia (3.30%), and depression (1.93%). Hyperlipidemia, Hypertension, and obesity affected 1.65%, 1.38%, and 1.38% of respondents, while 75.8% of patients reported no comorbid conditions. It should be noted that comorbidities such as anxiety and insomnia are commonly reported among patients with severe primary hyperhidrosis and are understood to often reflect the significant psychosocial burden of the condition.

**TABLE 1:** Baseline characteristics of enrolled outpatients (n = 363).

Item	Ratio
Gender	
Male	49.9% (181)
Female	50.1% (182)
Age (years, mean ± SD)	27 ± 8
Hight (cm, mean ± SD)	169 ± 8
Weight (kg, mean ± SD)	63.3 ± 12.8
Education	
Graduate	12.4% (45)
Undergraduate	57.8% (210)
Junior and senior high school	29.2% (106)
Primary school and below	0.55% (2)
Occupation	
Student	35.3% (128)
Industry	1.38% (5)
Construction	4.41% (16)
Transportation, postal and telecommunications	3.58% (13)
Commerce, public catering, materials supply, and storage	1.93 (7)
Real estate management, public utilities, residential services, and consulting services	1.65% (6)
Public health, sports, and social welfare	4.41% (16)
Education, culture and arts, and radio and television	4.41% (16)
Scientific research and comprehensive technical services	3.03% (11)
Finance and insurance	3.31% (12)
State organs, party and government agencies, and social organizations	3.86% (14)
Others	32.8% (119)
Comorbidities	
None	75.8% (275)
Hypertension	1.38% (5)
Diabetes mellitus	0.55% (2)
Hyperlipidemia	1.65% (6)
Insomnia	3.30% (12)
Anxiety	6.06% (22)
Depression	1.93% (7)
Obesity	1.38% (5)
Malnutrition	0.55% (2)
Pleuritis	0.28% (1)
Others	7.16% (26)

The disease burden characteristics are detailed in Table 2. The duration of PH was 17 [10–20] years, and 27.3% of participants reported a positive family history of hyperhidrosis. Based on self-assessed disease severity using HDSS, a substantial burden was observed: 87.3% of patients experienced frequent or constant interference with daily activities (HDSS grade 3–4), including 46.0% who rated their symptoms as “barely tolerable” (HDSS grade 3) and 41.3% as “intolerable” (HDSS grade 4). The most commonly affected areas were the hands (97.8%), feet (89.3%), and axillae (53.4%). Commonly reported sweating triggers included emotional tension (86.2%), high temperature (73.8%), anger (69.7%), and excitement (68.3%).

**TABLE 2:** Disease burden of hyperhidrosis among enrolled outpatients (n = 363).

Item	Ratio
Duration of PH (years, median[IQR])	17 [10–20]
Family history of hyperhidrosis	
Yes	27.3% (99)
No	50.1% (182)
Unclear	22.6% (82)
Self-assessment of degree of PH (HDSS scale)	
Grade 1	0.60% (2)
Grade 2	12.1% (44)
Grade 3	46.0% (167)
Grade 4	41.3% (150)
Affected areas of PH	
Hands	97.8% (355)
Axillae	53.4% (194)
Head and face	15.4% (56)
Feet	89.3% (324)
Chest and back	6.6% (24)
Abdomen	1.93% (7)
Buttocks	6.89% (25)
Legs	3.31% (12)
Reported sweating triggers	
High temperature	73.8% (268)
Emotional tension	86.2% (313)
Excitement	68.3% (248)
Dysphoria	48.8% (177)
Anger	69.7% (253)
No specific trigger	27.8% (101)

### Landscape of current hyperhidrosis management

**FIGURE 1:**
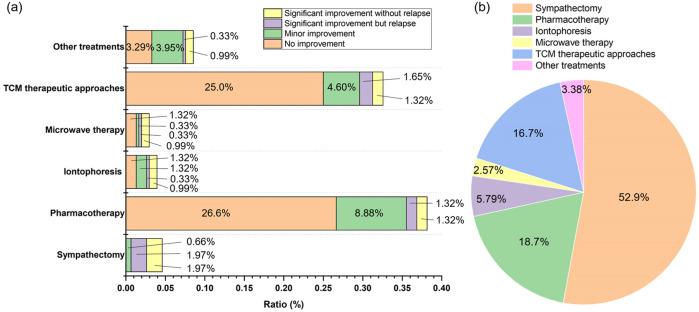
**(a)** History of treatments received and corresponding patient-reported efficacy following PH diagnosis (n = 304); **(b)** Level of awareness regarding available treatments for PH among the surveyed patient cohort (n = 363). It should be noted that TCM therapeutic approaches encompass TCM, acupuncture, massage and manipulation therapies.

All participants were inquired about treatments received since PH onset (Figure 1a). The proportions of patients who had undergone sympathectomy, iontophoresis, and microwave therapy were 4.60%, 3.96%, and 2.97%, respectively. In contrast, pharmacotherapy and Traditional Chinese Medicine (TCM) therapeutic approaches were used by 38.1% and 32.6% of participants, respectively. However, 25.0% of those who tried TCM therapeutic approaches and 26.6% of those receiving pharmacotherapy reported no symptomatic improvement, while only 4.60% (TCM therapeutic approaches) and 8.88% (pharmacotherapy) noted minor improvement. Evaluation of patient awareness regarding available treatments (Figure 1b) revealed that 52.9% were aware of sympathectomy. In comparison, awareness was lower for pharmacotherapy (18.7%) and TCM therapeutic approaches (16.7%). Knowledge of other interventions was limited, with only 5.79% aware of iontophoresis and 2.57% of microwave therapy.

**FIGURE 2:**
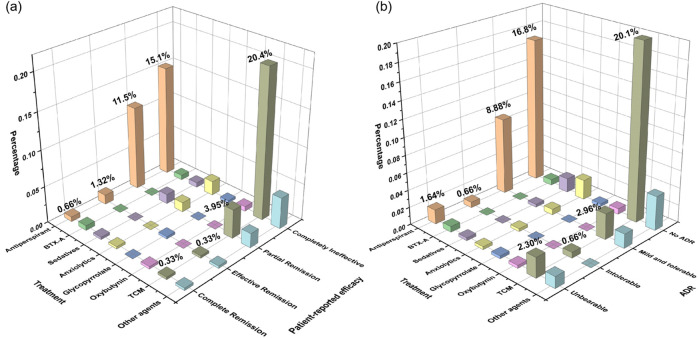
Patient-reported evaluation of **(a)** treatment efficacy and **(b)** adverse effects associated with previous pharmacological interventions among all respondents. The total effectiveness rates **(a)** of corresponding medication users are antiperspirant (47.1%, 95% CI [36.4, 57.8]), BTX-A (50.0%, 95% CI [-41.9,142]), sedatives (71.4%, 95% CI [26.3, 116]), anxiolytics (45.4%, 95% CI [10.4, 80.5]), glycopyrrolate (50.0%, 95% CI [-585, 685]), oxybutynin (33.3%, 95% CI [-110, 177]), TCM (18.4%, 95% CI [9.50, 27.3]), and other agents (43.3%, 95% CI [15.4, 60.8]). The total ADRs ratios **(b)** of corresponding medication users are antiperspirant (40.0%, 95% CI [29.4, 50.6]), BTX-A (50.0%, 95% CI [−41.9, 142]), sedatives (28.6%, 95% CI [−16.6, 73.7]), anxiolytics (36.4%, 95% CI [2.47, 70.3]), glycopyrrolate (50.0%, 95% CI [−585, 685]), oxybutynin (33.3%, 95% CI [−110, 177]), TCM (22.8%, 95% CI [13.3, 32.2]), and other agents (42.9%, 95% CI [19.8, 65.9]). BTX-A: botulinum toxin A (n = 304).

Figure 2 outlines the pharmacological treatments previously used, which included antiperspirant, BTX-A, sedatives, anxiolytics, glycopyrrolate, oxybutynin, TCM, and other agents. Evaluations of patient-reported efficacy (Figure 2a) and adverse drug reactions (ADRs, Figure 2b) indicated limited experience with pharmacotherapies other than antiperspirants and TCM. Antiperspirants were more commonly used than TCM and were associated with a higher rate of partial remission (11.5% vs. 3.95%). ADRs were reported by 11.2% of antiperspirant users, slightly higher than the 5.92% reported by TCM users. Notably, 1.64% and 2.30% of patients described ADRs from antiperspirants and TCM, respectively, as unbearable.

**FIGURE 3:**
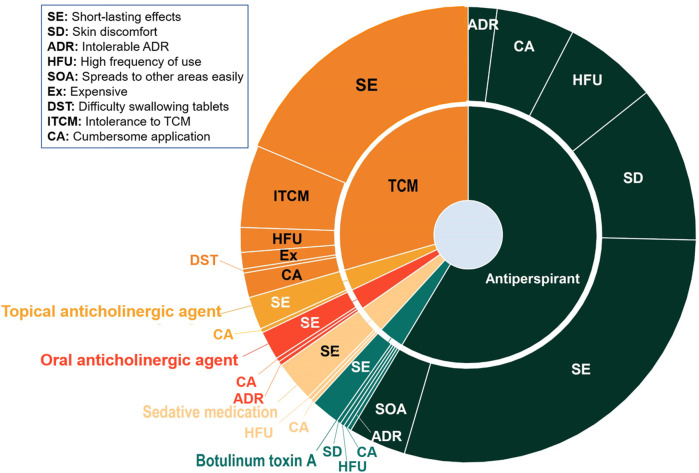
Patient-reported concerns associated with previously utilized pharmacological treatments (n = 363).

We investigated patient concerns regarding previous pharmacotherapy experiences (Figure 3). The most frequently reported issue across antiperspirants, BTX-A, sedatives, oral and topical anticholinergic agents, and TCM was short-lasting effects (SE). Antiperspirant users also frequently reported skin discomfort (30.9%) and inconvenient dosing frequency (18.7%). As most antiperspirants available in China are formulated as gels or creams, participants expressed dissatisfaction with cumbersome application (15.4%) and unintended transfer to unaffected areas (11.4%). For TCM, which primarily involves oral herbal decoctions, 23.0% of patients reported poor tolerability due to difficulty in consuming decoctions.

### Exploration of preferences for further pharmacotherapy

**FIGURE 4:**
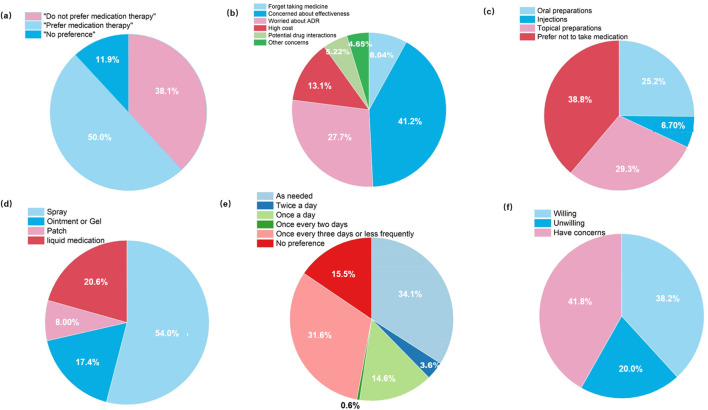
Patient preferences and perspectives regarding future pharmacotherapy: **(a)** Willingness to adopt pharmacotherapy if symptoms persisted or recurred after surgery; **(b)** Primary concerns associated with future drug therapy; **(c)** Preferred dosage forms; **(d)** Preferred application methods for topical formulations; **(e)** Preferred dosing frequency; **(f)** Willingness to participate in clinical trials investigating novel topical agents for PH (n = 363).

The survey yielded critical insights into the preferences of PH patients regarding future pharmacotherapy (Figure 4). Regarding postoperative pharmacotherapy willingness (Figure 4a), 50.0% of respondents were willing to use medication if symptoms persisted or recurred after surgery, whereas 38.1% were unwilling. Primary concerns related to pharmacotherapy (Figure 4b) included inadequate effectiveness (41.2%) and ADRs (27.7%), which outweighed considerations such as high cost (13.1%) or forget taking medicine (8.04%).

In terms of preferred dosage forms (Figure 4c), topical formulations were favored (29.3%), exceeding oral preparations (25.2%) and injections (6.70%). Furthermore, spray-based delivery systems were the overwhelming choice (54.0%), reflecting a demand for convenient and non-contact application (Figure 4d). Regarding dosing frequency (Figure 4e), as-needed dosing (34.1%) and regimens administered once every 3 days or less frequently (31.6%) were preferred over once-daily dosing (14.6%) or no preference (15.5%). Willingness to participate in clinical trials was also assessed (Figure 4f), 38.2% of respondents expressed openness to enrolling in trials investigating novel topical agents, although 41.8% reported unresolved reservations.

**FIGURE 5:**
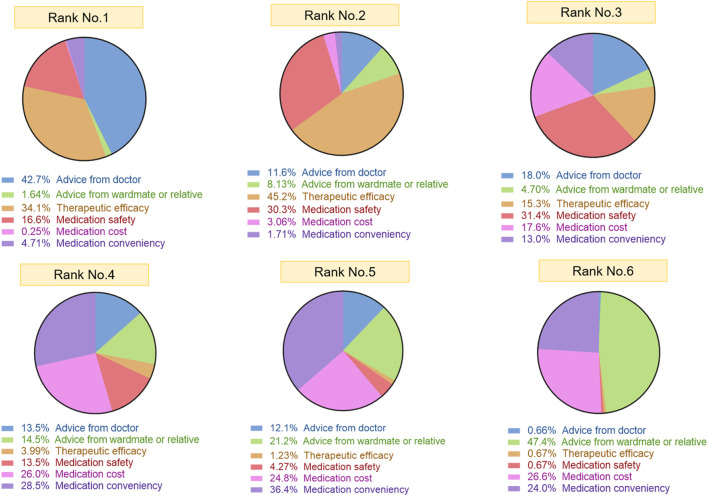
Hierarchical ranking of factors influencing treatment decision-making among patients with PH. “Rank No.1-6” refers to the ranking of important factors considered by the surveyed patients when choosing treatments. The chart for “Rank No.1” displays the first class consideration factor and its percentage among patients when receiving treatment, and the same logic applies to “Rank No.2-6” (n = 363).

Patients demonstrated a distinct hierarchy when prioritizing treatment attributes (Figure 5). Physician recommendation was the most frequently selected first-rank factor (42.7%), representing the predominant influence on therapeutic decision-making. Therapeutic efficacy emerged as the dominant second-rank consideration (45.2%), whereas safety concerns were most commonly prioritized as the third-rank factor (31.4%). Medication convenience was most frequently ranked as the fourth or fifth priority (28.5% and 36.4%, respectively). Advice from wardmate or relatives was most often assigned the sixth rank (47.4%), indicating a marginal influence. Overall, physician recommendation, therapeutic efficacy, and medication safety consistently constituted the foremost considerations influencing patients’ treatment choices.

### Analysis of factors influencing treatment regimen selection

We analyzed factors influencing the selection of treatment regimens among patients with PH, including education level, gender, age, and disease severity (Table 3). Pharmacotherapy was the most preferred treatment option in undergraduate outpatients (56.9%). While, iontophoresis was favored by patients at junior and senior high school level (41.7%). However, no significant differences in treatment preference were observed based on education level (all *P* > 0.05).

**TABLE 3:** Factors associated with the selection of treatment regimens (n = 304).

Factors		Sympathectomy	Pharmacotherapy	Iontophoresis	Microwave therapy	TCM therapeutic approaches	Other treatments
Education	Graduate	0	18.1%	33.3%	22.2%	16.2%	19.2%
	Undergraduate	78.6%	56.9%	25.0%	44.4%	58.6%	53.8%
	Junior and senior high school	21.4%	25.0%	41.7%	3.4%	25.2%	26.9%
	Primary school and below	0	0	0	0	0	0
	*P* value	0.311	0.075	0.083	0.825	0.474	0.809
	Cramér’s V	0.108	0.152	0.148	0.054	0.091	0.056
Gender	Male	50.0%	50.9%	83.3%	77.8%	51.5%	57.7%
	Female	50.0%	49.1%	16.7%	22.2%	48.5%	42.3%
	*P* value	0.980	0.737	0.020**	0.095	0.774	0.432
	Cramér’s V	0.001	0.020	0.134	0.096	0.016	0.045
Age	<18	0	6.90%	8.30%	0	6.10%	11.5%
	18–30	50.0%	68.1%	58.3%	55.6%	68.7%	61.5%
	>30	50.0%	25.0%	33.3%	44.4%	25.3%	26.9%
	*P* value	0.142	0.153	0.943	0.436	0.222	0.875
	Cramér’s V	0.113	0.114	0.020	0.074	0.100	0.030
Disease Severity (HDSS)	Grade 1	0	0	0	0	0	0
	Grade 2	14.3%	9.5%	25.0%	11.1%	7.1%	3.8%
	Grade 3	35.7%	46.3%	25.0%	22.2%	45.5%	42.3%
	Grade 4	50.0%	43.9%	50.0%	66.7%	47.5%	53.8%
	*P* value	0.837	**0.040** ^**#**^	0.442	0.384	**0.005** ^**##**^	0.237
	Cramér’s V	0.053	0.130	0.094	0.100	0.165	0.113

** indicates a statistically significant difference in the Pearson Chi-Square (P<0.01); # and ## represent statistically significant differences in the linear significant correlation (χ^2^_L) at P<0.05 and P<0.01, respectively. Bold type highlights significant P values.

Both male and female participants showed similar preferences for sympathectomy (50.0% vs. 50.0%), pharmacotherapy (50.9% vs. 49.1%) and TCM therapeutic approaches (51.5% vs. 48.5%). A statistically significant gender-based difference was observed in the preference for iontophoresis, with males more likely to select this option compared to females (83.3% vs. 16.7%, *P* = 0.020, OR = 0.192, 95% CI [0.041, 0.891]). No other significant gender-related differences were identified.

Pharmacotherapy (68.1%) and TCM therapeutic approaches (68.7%) were the most common choices across 18–30 age groups. A trend toward decreased preference for pharmacotherapy, iontophoresis, microwave therapy and TCM therapeutic approaches was noted with advancing age (18–30 age group vs. >30 age group), though this did not reach statistical significance. Patients with higher disease severity (HDSS Grade 4) showed a stronger preference for sympathectomy (50.0%), iontophoresis (50.0%), microwave therapy (66.7%) and TCM therapeutic approaches (47.5%). Disease severity was significantly associated with the likelihood of selecting pharmacotherapy (*P* of χ^2^_L = 0.040) and TCM therapeutic approaches (*P* of χ^2^_L = 0.005).

We performed binary logistic regression on the variables that showed a significant linear association in the chi-square test data (HDSS with pharmacotherapy and TCM therapeutic approaches). In the relationship of HDSS with pharmacotherapy, the Omnibus test of model coefficients yielded χ^2^ = 5.367, df = 3, p = 0.147, R^2^ = 0.025 indicating that, overall, HDSS and pharmacotherapy choice did not follow a significant linear pattern. However, the individual predictors in the equation revealed that Grade 2/Grade 4 exerts a significant effect on treatment selection (*P* = 0.043, OR = 0.446, 95% CI [0.204, 0.976]). In the relationship of HDSS with TCM therapeutic approaches, the Omnibus test of model coefficients yielded χ^2^ = 9.205, df = 3, *P* = 0.027, R^2^ = 0.042, indicating the overall significant linear pattern. Especially, Grade 2/Grade 4 exerts a significant effect on TCM therapeutic approaches selection (*P* = 0.008, OR = 0.298, 95% CI [0.122, 0.725]). It is understandable that the more pronounced the symptoms, the greater the likelihood of opting for pharmacotherapy and TCM therapeutic approaches.

### Impact of previous treatments on subsequent willingness for pharmacotherapy

The Spearman correlation coefficient between pre- and post-treatment HDSS scores was 0.908 (*P* = 0.000), indicating a strong positive correlation and suggesting that patients with more severe symptoms at baseline continued to experience significant symptom burden after treatment. This correlation underscores the suboptimal efficacy of current treatment approaches.

The overall multinomial logistic regression model was not significant (likelihood-ratio test: χ^2^ (12) = 15.63, *P* = 0.209), with a Nagelkerke pseudo-R^2^ of 0.059, indicating that the model explained approximately 5.9% of the variance in the dependent variable and achieved an overall prediction accuracy of 62.8%. Likelihood-ratio tests for individual predictors showed that, in terms of efficacy, prior experience with antiperspirant (χ^2^ (2) = 3.79, *P* = 0.150) and TCM (χ^2^ (2) = 2.23, *P* = 0.329) did not significantly influence subsequent willingness to undergo pharmacotherapy. Similarly, from the perspective of adverse drug reactions (ADR), prior experience with antiperspirant (χ^2^ (2) = 4.14, *P* = 0.126) and TCM (χ^2^ (2) = 0.50, p = 0.778) had no significant effect on future willingness to pursue pharmacological treatment.

As displayed in Table 4, no significant preference was observed between prior antiperspirants efficacy and subsequent willingness to pursue pharmacotherapy (Willingness vs. Reluctance, OR = 1.140 [0.595, 2.185], *P* = 0.692). A non-significant trend toward reduced pharmacotherapy willingness (Willingness vs. Reluctance, OR = 0.536 [0.226, 1.270], *P* = 0.156; Neutral preference vs. Reluctance, 0.615 [0.152, 2.483], *P* = 0.494) was noted among patients who had previously tried TCM. Adverse effects related to antiperspirant use did not significantly influence willingness to consider future pharmacotherapy (Willingness vs. Reluctance, OR = 0.957 [0.686, 1.335], *P* = 0.794). However, patients who experienced less severe side effects showed a marginal tendency toward neutrality (“no preference”) rather than outright reluctance (OR = 1.631 [0.941, 2.827], *P* = 0.081). Adverse effects associated with TCM did not exert a significant effect on future pharmacotherapy acceptance.

**TABLE 4:** Influence of previous treatments on willingness for future pharmacotherapy by multinomial logistic regression (n = 304).

Item	Pharmacotherapy	Willingness vs. Reluctance (OR)	*P* value	Neutral preference vs. Reluctance (OR)	*P* value
Therapeutic effect evaluation	Antiperspirant	1.140 [0.595,2.185]	0.692	0.346 [0.086,1.404]	0.138
	TCM	0.536 [0.226,1.270]	0.156	0.615 [0.152,2.483]	0.494
Adverse effects	Antiperspirant	0.957 [0.686,1.335]	0.794	1.631 [0.941,2.827]	0.081
	TCM	1.094 [0.807,1.483]	0.564	1.147 [0.717,1.834]	0.567

## Discussion

### The current treatment landscape for PH remains suboptimal

As this survey was conducted in a thoracic surgery outpatient clinic, the enrolled cohort was characterized by a high burden of disease, with the vast majority (87.3%) of participants self-reporting severe symptoms (HDSS Grade 3 or 4). A selection bias must be acknowledged. Most participants had extensively researched surgical treatments prior to consultation, with 52.9% aware of sympathectomy - a proportion likely significantly higher than that in the general hyperhidrosis population, occurred mostly during the youth years (Nawrocki and Cha, 2019a). Although pharmacotherapy represents a more accessible treatment option (Nawrocki and Cha, 2019b), public awareness of medicinal treatments for PH appeared limited (18.7%). TCM therapeutic approaches, a distinctive feature of China’s healthcare system, was frequently sought for constitutional regulation and symptom management (16.7%) (Youn et al., 2022).

Though guidance provides various treatment recommendations according to HDSS score (McConaghy and Fosselman, 2018), our survey revealed that pharmacotherapy (38.1%) and TCM therapeutic approaches (32.6%) were the most commonly tried modalities. In contrast, iontophoresis and microwave therapy showed limited adoption, the low uptake of these modalities is likely attributable, at least in part, to their dependence on specialized equipment and the need for repeated clinic visits, compounded by extremely low patient awareness (iontophoresis 5.79%, microwave therapy 2.57%), these factors that may jointly limit both accessibility and long-term adherence. Our analysis revealed higher utilization rates of antiperspirants and TCM; However, patient-reported efficacy was suboptimal. The majority of TCM users reported no therapeutic benefit, whereas 40.2% of antiperspirants users experienced partial symptom relief - compared to only 15.8% for TCM. Safety profiles were generally favorable for both, though antiperspirants were associated with a higher incidence of reported ADRs (40.0% vs. 22.8% for TCM). However, the patient-reported effectiveness of TCM should be interpreted cautiously. TCM management of PH emphasizes holistic ‘pattern-based diagnosis and treatment’ and long-term constitutional regulation; its therapeutic goals may extend beyond rapid, short-term sweat suppression to include alleviation of accompanying symptoms and re-balancing of systemic status.

### Desire and concerns regarding pharmacotherapy among PH patients

Building on prior medication experiences, patients identified short-lasting effects as the primary drawback of antiperspirants. Skin discomfort following topical application was another significant concern. For TCM, a considerable proportion of users reported difficulty tolerating its characteristic flavor. These findings underscore that efficacy, durability, convenience, and comfort are critical determinants of treatment decisions, highlighting the need for healthcare quality improvements that prioritize patient-reported experiences and tolerability (Mansfield et al., 2019).

We further examined key drivers of pharmacotherapy preferences. Half of patients (50.0%) expressed willingness to use pharmacotherapy, with efficacy and ADRs being their foremost concerns. Treatment cost also emerged as a significant factor. These priorities align with the core principles of pharmacotherapy: safety, efficacy, and cost-effectiveness. Additionally, patients favored convenience-oriented delivery systems, with 29.3% preferring topical preparations and 25.2% oral formulations - especially sprays - reflecting the need for user-friendly administration in hand-focused hyperhidrosis. Dosing frequency preferences included convenient use (34.1%) and applications no more frequent than every 3 days (31.6%), indicating that treatment burden substantially influences real-world adherence.

When ranking factors influencing medication choices, physician recommendations were the most influential (42.7% first-rank), underscoring the central role of clinicians in PH treatment decisions within China’s medical context (Han et al., 2022). Medication efficacy and safety were ranked second and third, respectively, consistent with our findings above. Price and convenience of medication use were less dominant, while recommendations from relatives or wardmates were least important - reinforcing patients’ trust in medical authority.

### Gender and disease severity as influencing factors of pharmacological treatment options

Our analysis identified several key factors shaping treatment preferences. The broad preference for pharmacotherapy across demographics suggests its perceived convenience. However, the lack of significant *P*-values for education, gender and most age groups imply that socioeconomic factors may not be primary drivers. The higher preference for iontophoresis among males (*P* = 0.020) may reflect gender-based differences in tolerance for unconventional treatments or cultural perceptions of acceptability.

Patients’ choice of sympathectomy slightly increased with age, indicating a more conservative attitude toward surgery among older patients, though this trend was not statistically significant. Surgery is generally not recommended as first-line treatment for individuals under 18, and no adolescents in our survey population had undergone surgical intervention (Cerfolio et al., 2011). While some patients may experience symptom reduction with age, older patients tended to prefer surgery - possibly due to more severe or prolonged symptoms, or a stronger desire for definitive relief.

The preference for pharmacotherapy and TCM among patients with higher HDSS grades aligns with the need for aggressive management in severe cases. The significant preference for TCM (*P* = 0.005) further suggests that patients with severe symptoms may seek complementary treatment options.

### Prior treatment experiences show limited impact on pharmacotherapy willingness

We also evaluated how prior treatment experiences influenced patients’ willingness to pursue pharmacotherapy. The lack of significant associations between previous treatment efficacy and future pharmacotherapy willingness indicates that patients do not base decisions solely on prior outcomes. Although not statistically significant, TCM users showed a trend toward lower willingness to try pharmacotherapy, possibly reflecting a preference for non-pharmacological approaches among some subgroups. The fact that ADRs did not strongly deter pharmacotherapy willingness suggests that symptom control outweighs side-effect concerns in chronic conditions like hyperhidrosis. A marginally neutral stance (OR = 1.631 [0.941, 2.827], *P* = 0.081) among antiperspirant users who experienced side effects may indicate hesitancy toward TCM-based pharmacotherapy.

Since prior treatment experiences did not strongly predict pharmacotherapy willingness, clinicians should proactively assess patient preferences rather than assuming reluctance based on historical therapies. The neutral attitude among some patients - especially those with prior ADRs - suggests that enhanced patient education on available options could support more informed decision-making.

## Limitations

This study has several limitations. First, as a single-center, cross-sectional survey conducted in the thoracic surgery outpatient clinic of a top-tier hospital, our sample mainly comprised patients with severe disease (Grade 3 and 4) and a clear surgical intention, resulting in obvious selection bias; consequently, the findings may not be fully generalizable to community-based outpatients with milder symptoms or those who are hesitant about surgery. Second, all data were based on patient-report and may therefore be subject to recall bias (especially for treatments received many years ago) and social-desirability bias (e.g., patients might tend to report more severe symptoms or greater treatment willingness to justify their visit). Although we attempted to minimize the latter by administering the survey anonymously, its influence cannot be completely ruled out. Finally, although the questionnaire used in this study underwent expert review and pilot testing, it has not yet been validated in a large sample to establish its construct validity, which may introduce some measurement error.

Future studies should adopt a multicenter design, include a broader patient population, and employ assessment tools that have undergone rigorous reliability and validity testing to further corroborate our findings.

## Conclusion

Although this survey was limited to outpatients in the thoracic surgery department of a single institution, it offers valuable insights into the current treatment landscape for PH patients, particularly those with severe symptoms. The overall treatment situation remains suboptimal, with over half of patients having never received any intervention. Among treated patients, pharmacotherapy and TCM therapeutic approaches were the most common modalities, yet patient-reported efficacy was unsatisfactory, highlighting an urgent need for more effective and safer pharmacological options. Efficacy and safety remained patients’ primary considerations when selecting drug treatments, with physicians’ recommendations playing a decisive role. Gender and disease severity may influence treatment preferences, but prior unsatisfactory experiences did not significantly diminish willingness of pharmacotherapy, indicating substantial unmet demand for effective drug-based treatments. Taken together, the patient preferences uncovered by this study, namely, a demand for topical dosage forms, longer dosing intervals (≥3 days), and higher efficacy coupled with a reassuring safety profile, chart a clear roadmap for future drug development. The significant gap between treatment needs and current status of PH in China suggests considerable potential for further development and research in PH therapeutics.

We also realize that future investigations must adopt a multicenter design and recruit patients from diverse settings such as dermatology and general-practice clinics, so that a comprehensive picture of the full spectrum of palmar hyperhidrosis in China can be obtained.

## Data Availability

The original contributions presented in the study are included in the article/Supplementary Material, further inquiries can be directed to the corresponding author.
