# Supplementary figures and images for: A significant gap between inadequate pharmacotherapy and substantial unmet needs in palmar hyperhidrosis management in China: insights from a questionnaire-based survey among outpatients
Source: Front Pharmacol. 2026 Jan 8;16:1715189. doi: 10.3389/fphar.2025.1715189 (PMC12823788; doi:10.3389/fphar.2025.1715189)

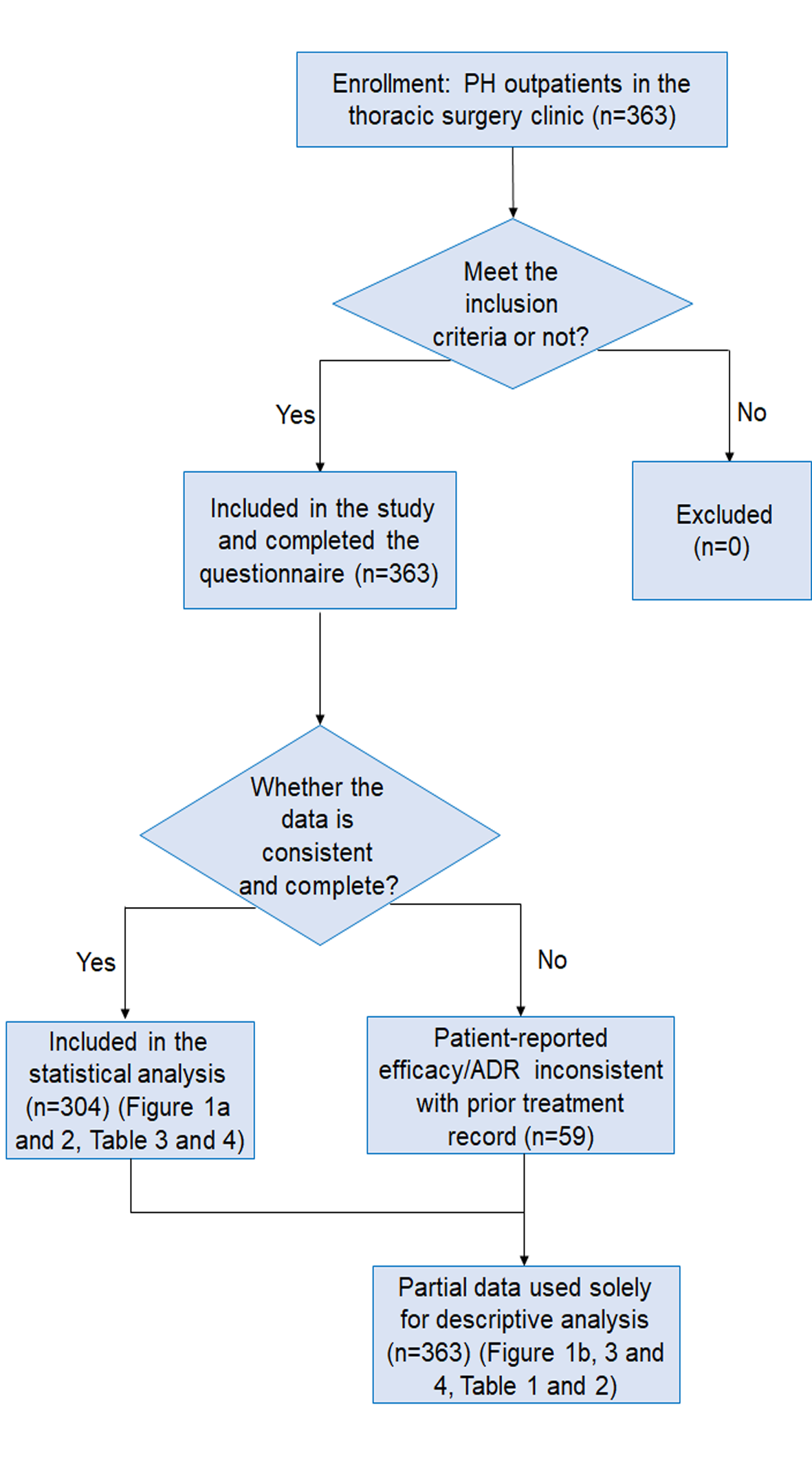

Supplement: Supplementary file 1 [file Image1.tif]

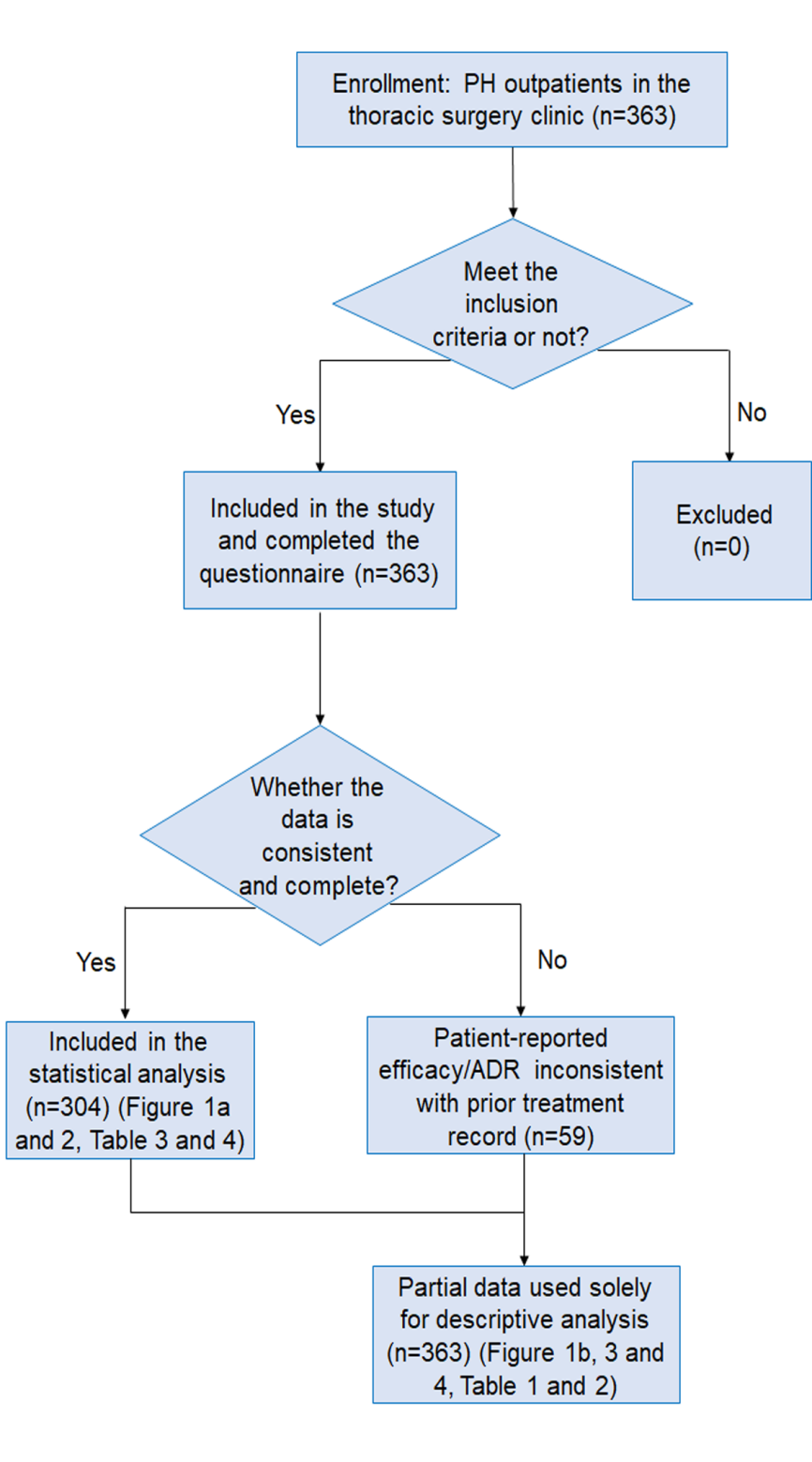


Supplementary Figure 1. Participant Flowchart of our study

Supplement: Supplementary file 2 [file DataSheet1.docx]
